# A Practical Approach to the Analysis and Optimization of Neural Networks on Embedded Systems

**DOI:** 10.3390/s22207807

**Published:** 2022-10-14

**Authors:** Mario Merone, Alessandro Graziosi, Valerio Lapadula, Lorenzo Petrosino, Onorato d’Angelis, Luca Vollero

**Affiliations:** Research Unit of Computer Systems and Bioinformatics, Department of Engineering, Universitá Campus Bio-Medico di Roma, Via Alvaro del Portillo, 21, 00141 Rome, Italy

**Keywords:** Internet of Things, edge computing, artificial intelligence, embedded system, convolutional neural network, optimization method, crowd counting

## Abstract

The exponential increase in internet data poses several challenges to cloud systems and data centers, such as scalability, power overheads, network load, and data security. To overcome these limitations, research is focusing on the development of edge computing systems, i.e., based on a distributed computing model in which data processing occurs as close as possible to where the data are collected. Edge computing, indeed, mitigates the limitations of cloud computing, implementing artificial intelligence algorithms directly on the embedded devices enabling low latency responses without network overhead or high costs, and improving solution scalability. Today, the hardware improvements of the edge devices make them capable of performing, even if with some constraints, complex computations, such as those required by Deep Neural Networks. Nevertheless, to efficiently implement deep learning algorithms on devices with limited computing power, it is necessary to minimize the production time and to quickly identify, deploy, and, if necessary, optimize the best Neural Network solution. This study focuses on developing a universal method to identify and port the best Neural Network on an edge system, valid regardless of the device, Neural Network, and task typology. The method is based on three steps: a trade-off step to obtain the best Neural Network within different solutions under investigation; an optimization step to find the best configurations of parameters under different acceleration techniques; eventually, an explainability step using local interpretable model-agnostic explanations (LIME), which provides a global approach to quantify the goodness of the classifier decision criteria. We evaluated several MobileNets on the Fudan Shangai-Tech dataset to test the proposed approach.

## 1. Introduction

Nowadays, Internet of Things (IoT) encompasses many areas of modern life, thanks to the widespread adoption of smart devices, able to receive, record, and send data. However, the large amount of data collected requires the proper technologies to record and process it. It is precisely to address these needs that we are now seeing more and more solutions exploiting artificial intelligence within IoT solutions to support and enable data processing. Moreover, the improvement of embedded systems, i.e., combination of hardware and software designed for a specific function, has led them to represent one of the key solutions to face the problems generated by the large amount of sensors generated data [1]. These systems can enable the implementation of a decentralized approach, shifting calculations closer to the data sources, this kind of approach takes the name of edge computing [2]. The adoption of the abovementioned solution enables better scalability of architectures by avoiding Cloud abuse and reducing dependence on the availability of an Internet connection [3], consequently reaching lower overheads and powers requirement if compared to large data centers. Furthermore, they boast robustness to attacks and improve information processing security and transparency in data treatment, giving privacy by design thanks to the brief need for data transfer. Therefore, limiting, if not eliminating, one of the most vulnerable phases to external attack. Edge computing also overcomes the problem of the side-real distance that data centers can have from the acquisition source. Think of all the control systems in the automotive [4,5,6,7] and industrial robotics sectors [8]: they need instantaneous implementation commands, for which response time must be minimized. The decentralized approach provides a twofold reduction in latency times [9], on one hand, due to less traffic in the network, and on the other hand because of the greater proximity between the decision layer and data location. In parallel, the growth of deep learning has recently taken AI to the next level, especially in several branches of computer vision, and fostered the development of high-performance edge computing systems that would support the new Deep Neural Networks (DNN) algorithms. However, the embedded system’s hard constraints and the typical complexity reachable by DNNs involve the need to discover the best algorithm to be ported to the edge device. Often, even starting from the best solution, there is the necessity to optimize the algorithm and lighten the computational load to increase performance and make the choice competitive. Nevertheless, several tasks wherein AI and embedded systems meet each other are worth mentioning: detection of abnormal events [10], such as people aggression, thefts, and objects loss; characterization of human gait [11]; congestion analysis; wireless networks security [12]; people identification; geo-textual hierarchical clustering [13]; pedestrian detection in urban scenes; etc. Moreover, in the field of NN recent studies are stating that it is not enough to know the performance of a classifier: even those with excellent performances can mislead about what they have learned [14]. Therefore, explainable artificial intelligence (XAI) is a hot topic nowadays because it can help us in: choice justification, supervised verification, and changeability, among others.

This paper extends our previous work [15], and it presents a standardized optimization method for porting DNN to embedded devices, which is valid regardless of the AI classification task, algorithm pipeline, and board type. The universality of the proposed method stems from the assumption that there is no DNN suitable to solve any classification problem and different DNN adapt differently to different microcontrollers. In particular, our method is based on three steps:

1. Best trade-off step: it helps to search for the best NN solution between different pipelines for a specific board in use.
2. Optimization step: starting from the NNs identified in the previous step, it evaluates different optimization techniques searching for further improvements.
3. Explanation step: evaluates the classification criteria correctness for the task at hand with LIME interpretability algorithms.

The application task to pragmatically utilize the proposed method is the detection of crowded states. Historically, this problem was approached with regression [16], detection [17,18,19], or density estimation methods [20], but the best results in terms of performance have been recently obtained with the advent of convolutional Neural Networks [21,22]. To date, the state of the art related to the crowd counting problem has always focused unilaterally on performance aspects, while few edge implementations emerge.

The paper is organized as follows: Section 2 presents the related works and discusses the main optimization techniques. Subsequently, Section 3, starting from the constraints derived by the implementation of DNN on the embedded system, goes on to describe all the choices: board and NNs to use, the case study and its coherent dataset to train the Neural Networks, and the adopted explanation algorithm. Section 4 describes the whole steps of the proposed method itself in detail plus the last subsection for the class weights method applied during the training phase. The results and associated discussions are shown in Section 5, divided into subsections such as the Methods section. Lastly, Section 6 reports further analyses and concludes.

## 2. Related Works

The growing interest in embedded solutions equipped with AI deep learning algorithms has led to the definition of specific compression and acceleration techniques for the prediction phase. The aims are to strictly adhere to the real-time constraints and to decrease the boards’ overloads. Below, we present a review of the main approaches and techniques.

As reported in [23], pruning is the first type of model reduction, based on the evaluation of the parameter’s importance. This method removes parameters identified as unimportant subject to classification performance constraints and optimizes the reduced Neural Network to maximize performance. Two types of pruning exist: (i) non-structured, in which Neural Network regularity and sparsity are not taken into account resulting in cache and memory access inefficiency; and (ii) structured pruning in which Neural Network regularity is an additional constraint.

The non-structured pruning approaches provide interesting performance, especially in terms of compression. In [24], Han et al. propose an iterative pruning technique for all the weights under a certain threshold after which retrain and optimize the remaining Neural Network. This method reduces memory requirements with a compression factor equal to 9. In [25], Guo et al. propose an approach that they call dynamic Neural Network surgery. This method allows recovering a critical but pruned connection based on a post-cut evaluation. The compression factor of this method reaches 17.7. Eventually, Li et al., in [26], manage to achieve a compression factor equal to 82 by restating the threshold tuning problem into a constrained optimization problem.

The structured approaches have slightly reduced compression gains when compared to the non-structured ones, but they usually couple Neural Network compression with better prediction and acceleration. In [27], Liu et al. use L1 regularization on the scaling factor of the Batch Normalization layers to train compact Neural Networks, reaching a compression factor and a prediction acceleration equal to, respectively, 6.6 and 1.43, without any drop in the top-5 accuracy value. Conversely, ThiNet, presented in [28], prunes filters by using the next layer statistics information instead of the current layer, obtaining 16.63 compression, 3.3 acceleration, and 0.52% top-5 accuracy drop for the VGG-16 net.

A second technique to reduce NNs size is quantization: this can concern weights, activations, or both. Among the weights quantization techniques, the Binary Connect (BC) [29] uses only two possible weights: −1 and +1. Tests on the ImageNet dataset show a 19.2% top-5 accuracy drop, but with a compression factor equal to 16 for the weights and a significant speed-up due to the complete removal of any multiplication. Moving from BC, the Binary Weight Network (BWN, [30]) better approximates the Neural Network to the starting CNN through a scaling factor, showing an improvement in the top-5 accuracy, which drops only by 6.2%, and slightly worsening in terms of memory and speed. The Binarized Neural Network (BNN, [31]) extends quantization to both weights and activations. It shows a 29.8% top-5 accuracy drop and a drastic decrease in memory usage. Similarly, the XNOR-Network [30] is an extension of BWN: it leverages the binary operations to approximate convolutions, achieving a 58 speed up in classification times and top-5 accuracy drops equal to 11% and 16% for AlexNet and ResNet-18, respectively.

A third Neural Network reduction technique is model distillation, whose aim is to transfer knowledge from a larger Neural Network (teacher) to a smaller distilled one (student). This approach finds a first excellent application in Noisy Teacher, where Sau et al. [32] propose a noise-based regularizer to improve the performance from 99.03% (teacher) accuracy to 99.14% (student) on MNIST dataset. In another study, Romero et al. [33] propose FitNets to train a thinner and deeper model on CIFAR-10 dataset. The student net reaches 91.63% accuracy from 90.18% of its teacher, decreasing from 9 to 2.5 million parameters.

Other techniques require the implementation of specific Neural Network strategies, such as the application of depthwise and pointwise convolutions, such as [34] on MobileNets, and filters size and number reduction, such as [35] on SqueezeNets. Lastly, the low-rank factorization technique is used to accelerate NNs by finding an approximate low-rank tensor (or matrix) close to the starting tensor and easier to decompose [36,37].

## 3. Materials

Unlike Personal Computers, typically, microcontroller boards rely on light architectures, tuned to perform fewer and application tailored-tasks. This is due to size and power constraints that translate into memory and computational limits. Indeed, microcontrollers usually have: (i) small Flash memory to host data (few MBs), (ii) small RAM memory (few hundreds kBs), (iii) reduced clock speeds (hundreds of MHz), and (iv) small data bus width (8/16 bits). An important aspect to consider is the power-to-performance ratio (typically in the tens of μW/MHz range). Indeed, power consumption and processing power are both critical factors for algorithm designers, especially when the goal is to avoid the overloading of embedded systems. From this point of view, it is important to have a trade-off between power consumption and algorithms complexity.

The offline deployment of NNs on embedded systems requires a two steps process: first, it requires defining and training the NN on a Workstation and, then, tuning the trained Neural Network on the chosen microcontroller. The design must take into account the capabilities of the physical device on which the Neural Network will be deployed. The constraints to deal with are the following:

(1) $$\begin{array}{ccc} {\mathrm{ROM}}_{\mu C} & > & {\mathrm{Parameters}}_{\mathrm{NN}} \end{array}$$

(2) $$\begin{array}{ccc} {\mathrm{RAM}}_{\mu C} & > & {\mathrm{Activations}}_{\mathrm{NN}} \end{array}$$

(3) $$\begin{array}{ccc} \frac{{\mathrm{NNcomplexity}}_{\mathrm{FLOPS}}}{{\mathrm{MCU}}_{\mathrm{FLOPS}}/s} & < & \frac{\mathrm{samples}\;\mathrm{in}\;a\;\mathrm{window}}{f_{\mathrm{sensor}\;\mathrm{sampling}\;\mathrm{rate}/s}} \end{array}$$

Equation (1) constrains the number of NN parameters to load in the available ${\mathrm{ROM}}_{\mu C}$ storage capacity of the read-only memory of the microcontroller. Equation (2) limits the number of activations and depends on the amount of available RAM. When managing DNNs, the layers that impact the most on RAM are the convolutional ones. Hence, a trade-off arises between classification performance, which depends mostly on the number of convolutional layers, and time performance, which may suffer the memory overload. Equation (3) represents the constraint for real-time applications: the ability to process the data on time and without losses.

Above inequalities can be rewritten as:

(4) $$\begin{array}{ccc} {\mathrm{ROM}}_{\mu C} & > & {\mathrm{Weights}}_{\mathrm{NN}} \end{array}$$

(5) $$\begin{array}{ccc} {\mathrm{RAM}}_{\mu C} & > & {\mathrm{Activations}}_{\mathrm{NN}} \end{array}$$

(6) $$\begin{array}{ccc} T_{\mathrm{inference}} & < & T_{\mathrm{Acquisition}} \end{array}$$

It is worth noting that these constraints depend exclusively on the NN architecture. When porting Neural Networks on embedded systems, however, we also need to consider additional burdens, such as the memory needed in pre- and post-processing phases (input and output Neural Networks buffers) and the execution of other concurrent programs. These constraints, combined with the growing need for edge implementations, have increasingly pushed the search for high-performance optimization solutions. However, as often happens, optimization leads to an even greater degree of abstraction, leading to a trust decline that users pour on models. For this reason, it is necessary to combine the explainability of the model with the optimization process.

In the next sections, we present the edge system, the Neural Network, and the application chosen for the study.

### 3.1. Edge System

In this work, we choose the STM32F767ZIT6U to develop our embedded system prototypes. We based our choice on the presence of a state-of-the-art microcontroller with excellent processing capabilities, and wide availability of peripherals for industrial applications with sufficient memories for NN porting and execution. The main characteristics of the device are:

- 2 MB of Flash Memory;
- 512 kB of static RAM;
- Max $f_{clock}$ = 216 MHz.

In the study, we set the maximum clock speed in all the tests.

We used STM32CubeIDE for software development and to interact with the device. STM32CubeIDE is an open-source IDE and toolchain system which allows, among the others, to inspect ST Microelectronics μCs and assess in advance, before code assembly, portability issues related to memory requirements. For each supported Neural Network, we evaluated its main deployment characteristics: ROM, RAM, Multiply and Accumulate operations (MACs), and quantity of parameters. We dissected the analysis of the total RAM into three components: input, Neural Network, and output RAM. These components refer to the Neural Network input layer, the hidden layers, and the Neural Network output layer, respectively. We trained and optimized all the models considered in this work on a workstation. We verified NN instances on both the embedded system and the workstation to obtain a deep insight into the parameters and the configurations impact on performance.

### 3.2. Neural Networks

In this work, we focused on MobileNets [34], whose full model is reported in Table 1. This Neural Network class implements an architecture with highly efficient convolutional layers called depthwise separable convolutions, and this makes them highly optimized for embedded and mobile applications.

DepthWise separable convolutions make use of two hyperparameters with a value in the range [0, 1], the width multiplier α, and the resolution multiplier ρ. The former controls the number of channels or channel depth, whereas the latter controls the input image resolution. We trained the MobileNets in full and shallow versions, where the shallow version reduces the number of consecutive convolutional layers from 5 to 1, varying their characteristic parameters α and ρ, and considering only RGB images.

### 3.3. Application Choice

The method presented in Section 4 will also evaluate the algorithm’s performance aspects. Therefore, it is necessary to choose a practical application as an example, without which it would be possible to analyze only the computational parameters. However, we emphasize the method’s independence from the application. The paper’s purpose is to define an approach and not to provide a specific case study.

The application example refers to crowded and uncrowded state classification in video surveillance contexts and social monitoring. Two main reasons have led to this choice: the first is related to porting since the classification task represents a versatile approach for a computer vision problem in an embedded environment. The second is an interpretability reason: treating crowd detection as a classification task can conduct different Neural Network learning logics. LIME, which is the method’s third part, can highlight these training phenomena and hopefully explain some Neural Network performance behaviors. Relating to the example state of the art the best results in terms of performance have been recently obtained with the advent of convolutional Neural Networks [21,22].

According to this, to address the problem we trained, validated and tested several MobileNets versions [38] on Fudan Shanghai Tech dataset [21]. The dataset includes 15,000 samples of 100 different scenes between indoor and outdoor environments, randomly divided into two sets: a training set of 9000 samples and a test set of 6000 samples. The criteria behind this particular dataset choice were the following:

- High number of samples;
- Average people per image consistent with typical values for indoor and outdoor workplace video surveillance systems;
- Excellent images starting resolution;
- Images from different contexts being able to represent a factor in favor of net generalization ability in the prediction phase.

Concerning the training phase, the classifier’s state division threshold was set to 20 people, maintaining a conservative approach between scene volumes and instances quantity for the image. Therefore, an unbalanced binary problem has been addressed, with a training set consisting of 2230 samples belonging to class 1 (crowd less than 20 people) and 6770 of class 2 (crowd greater than 20 people).

## 4. Methods

The propose method is divided into three steps. Firstly, in the Section 4.1, we present the best trade-off step. In this phase, information is extracted from the NN, e.g., RAM, ROM storage memory, and compared with the constraints due to the device itself. Secondly, in Section 4.2 the optimization step is explained. In this step, we consider three different approaches: regularization, pruning, quantization, and their possible combined use, so that the computational burden can be reduced and the inference time can be improved without degrading the goodness of the models. Finally, in Section 4.3 we present a possible approach to explain the logic behind model decisions, an operation that becomes even more important as a result of the network modifications made in the optimization step.

### 4.1. Best Trade-Off Step

In the first of the method three steps, ROM and RAM memories, MACs operations, and the number of parameters were extracted for each NN structure. By referring to the first two equations discussed in Section 3, Neural Networks that do not fit the board’s limits in terms of total RAM, ROM, or both, were removed.

Once obtained, the only implementable ones, to assess both performance and computational burden factors, the Neural Network choice was based on the best trade-off between accuracy, $T_{inference\;board}$, and loss factor β.

To burst these parameters, we conventionally indicate as Py-Net the NN implemented in Python on the workstation, whereas we indicate as C-Net the NN loaded on board and so implemented in C language.

As accuracy values, Py-Net performances on the entire test set were used, since, in the preliminary assessment, the relative error-index L2 between the Py-Net (Full Neural Network) output layer and the C-Net one never exceeded the critical value of 0.01 (cross-validations based on external test sets always returned 100% accuracy). Relating to the example, to achieve better accuracies and address the unbalanced problem, the class weights method was applied during the training phase, correlating weights to sample distribution, as in Equation (7).

(7) $$Class{\;}_{i}\;weight\;=\;\frac{1}{N\;classes}\;\times \;\frac{Total\;samples}{Class{\;}_{i}\;samples}$$

Concerning the other two best trade-off parameters, to understand networks behaviors either on a single image or on streams of images (up to 100 frames), different smaller random test sets of 1, 10, 50, and 100 images were created. When a test set shows multiple images, every frame has its time inference. Over these test sets, the $T_{inference\;board}$ in terms of means ($\bar{T_{inference\;board}}$ [ms]) and standard deviations ($\sigma_{\;\bar{T_{inference\;board}}}$ [ms]) was analyzed. Mean and standard deviation values were carried out for each implementable NN on all test frames and on all test sets. According to this, we expressed $T_{inference\;board}\;=\;\bar{T_{inf\;board}}\;\pm \;\sigma_{\;\bar{T_{inf\;board}}}\;\left[\mathrm{ms}\right]$, $T_{inference\;pc}\;=\;\bar{T_{inf\;pc}}\;\pm \;\sigma_{\;\bar{T_{inf\;pc}}}\;\left[\mathrm{ms}\right]$, and the loss factor $\beta \;=\;\frac{T_{inference\;board}}{T_{inference\;pc}}$ as $\beta \;=\;\bar{\beta }\;\pm \;\sigma_{\;\bar{\beta }}$.

In this work, we focused on RGB images to better exploit database characteristics and the capabilities of the chosen LIME explainers. However, a similar investigation also on grayscale images is conducted in [15].

Two more steps refine the best trade-off. The former is a canonical optimization step based on regularization, pruning, and quantization techniques to improve Neural Network speed and system resource utilization. The latter aims to highlight the superpixels that mostly influence the classifier decision-making for each image, and quantify the global goodness through the Intersection Over Union parameter.

### 4.2. Optimization Step

In this step, the aim was to investigate the impact of several mechanisms to improve Neural Network performances and understand the sensitivity of parameters to the different optimization techniques. To obtain a proper real-time application, both performance and computational parameters were analyzed: accuracy and time inference represent performance metrics, whereas RAM, ROM, and MACs represent computational metrics. Furthermore, we analyzed the impact of the number of the Neural Network on all metrics.

Among all the possible ways to optimize a Neural Network [23], three different approaches were considered:

- Regularization;
- Pruning;
- Quantization.

Regularization and Pruning were executed in parallel, then the resulting nets from the previous optimization steps were quantized as follows below. The first approach is related to specific regularization attempts applied on the best trade-off to enhance performances. Specifically, the three considered regularization attempts are:

1. Attempt R1: addition of a L2 regularization penalty = 0.0005 on layer 17 (“dense” with kernel regularizer).
2. Attempt R2: addition of L2 regularization penalty = 0.001 on layer 17 (“dense” with kernel regularizer).
3. Attempt R3: addition of L2 regularization penalty = 0.005 on layer 17 (“dense” with kernel regularizer) and L2 regularization penalty = 1 $\;\times \;\;10^{-5}$ on layer 15 (last depthwise convolution with activation regularizer on reLUs).

For R1, the L2 value equal to 5 $\;\times \;\;10^{-4}$ on weights has been chosen by considering Alex Krizhevsky, et al. [39] observation, according to which small-weight regularizations for convolutional Neural Networks act not only as such but even as learning improvers. Additionally, Simonyan et al. [40] used 1 $\;\times \;\;10^{-5}$ as weight decay to develop a CNN for the ImageNet dataset, founding this a promising quantity. Following Reed et al. [41], a 0.001 value has been chosen and selected between the good tests for R2. Finally, R3 includes both weight and activation L2 techniques and was dictated by the positive result.

The second approach is a pruning method that deletes sequentially, first, and then simultaneously the Neural Network’s bottlenecks in terms of time inference. Indeed, we profiled layer-by-layer different parameters, among which also the time inference. Five pruning attempts have been performed:

1. Attempt P1: the value stride is equal to 2 on layer 15, used in all subsequent tests and intended to lighten the dimensional change on pooling.
2. Attempt P2: removal of layers 9–10.
3. Attempt P3: removal of layers 5–6.
4. Attempt P4: removal of layers 5–6 and 9–10.
5. Attempt P5: removal of layers 0–1, 5–6, and 9–10.

The last approach is an 8-bit quantization which aims to evaluate how pixels, weights, and layer outputs representation reduction from 32 to 8 bits influences the global and layer-by-layer effectiveness in terms of all parameters. In practice, the quantization has been applied on all the Neural Networks resulting from the pruning and regularization. This approach required different steps including:

- The creation of a Keras “quantization aware model” from the starting Neural Network, to partially rebuild Neural Network architecture in order to facilitate the following steps.
- The training and validation of a quantization-aware model. It is worth noting that this Neural Network topology is not quantized yet.
- The conversion from Keras retrained quantization aware model (.h5) to the TensorFlow Lite model (.tflite).
- The evaluation of TensorFlow Lite model times and results.

We have to observe that quantization and pruning have a unique strategy approach, passing from 32 to 8 bits and starting from layer-by-layer analysis, respectively. Differently, regularization depends strictly on the application, so even if it is included as a technique within the method it does not have a unique way of implementation. As mentioned above, the method’s second step, composed of all the techniques discussed above, is essential to improve the real-time application in edge trying to minimize any kind of performance loss. It represents a reference step particularly significant to all the borderline cases where a starting Neural Network cannot be ported to the edge because of its excessive memory usage, allowing one to estimate and assess the goodness of alternative solutions.

### 4.3. Explanation Step

There are several approaches to explain the logic behind model decisions. In this study, Local Interpretable Model-Agnostic Explanation (LIME) [14] has been used. This algorithm processes the image to be explained and the model prediction on this image. Then, it divides the image into superpixels, and analyzing the local decision boundary, returns as output the most influencing superpixels in conditioning the decision in a specified class. In practice, the algorithm’s output is a binary mask of important superpixels.

In order to better understand the optimization steps impact, the LIME interpretability algorithm was exploited, applying it to the same 500 test samples used in the accuracy evaluation. The LIME algorithm creates an explainer for each image, and it returns an explanation for the specific frame. This latter is a mask that identifies the most relevant superpixels of the frame, those contributing the most to the correct classification outcome. From these masks, we computed the IOU (Intersection Over Union) coefficient for each test image, processing the masks of the original NN and that of the simplified one. After that, we investigated if accuracy and IoU trend lines correlate to demonstrate whether the optimization process affects the decision criterion.

## 5. Results and Discussions

### 5.1. Best Trade-Off

Table 2 reports the behavior of every RGB MobileNet in terms of the principal investigation parameters. A complete analysis is shown in [15]. Among all these nets only two (described in black on Table 2) are portable on the chosen edge device, respecting all memory constraints. Hence, the best trade-off choice between these is represented in Table 3: the two accuracy values are similar, with a small superiority of the deep model, but shallow structure shows, simultaneously, a time inference of 145.38±0.740 [ms] and a β factor of 2.213±0.070 lower than those demonstrated by the same deep version. The mandatory hard real-time constraint guided the shallow model as the best trade-off for this study, despite the small performance leak.

### 5.2. Optimization

**Figure 1:**
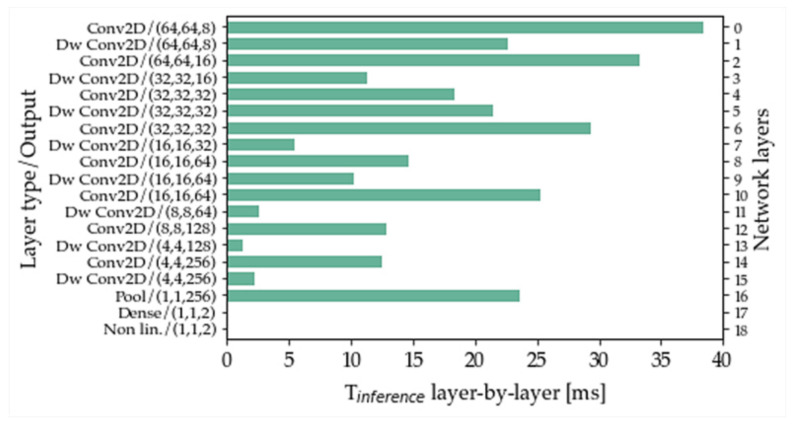
RGB Best trade-off: time inference layer by layer.

We analyzed and investigated parameter behavior layer by layer. This was helpful to understand on which layers RAM and ROM memories reside and where MAC operations are carried out in greater numbers, but especially to denote the best trade-off time inference layers bottlenecks. Figure 1 illustrates this latter aspect: the horizontals bars represent the layers times inference on board from the first C net layer (with “0” index) to the last nineteenth (with “18” index), compiled from up to down on the right y-axis. The left y-axis specifies the type and output dimensions of each net layer: the first layer type is a 2D standard convolution with (64 × 64 × 8) filters, while the last layer type is non-linearity and its output is a double neuron as binary classification expects.

In the Optimization Step section, specific pruning tests have been listed. The reason for these specific executions can be explained by observing Figure 1 itself. Firstly, confirmation takes place: the layer time inference strictly depends on input and output dimensions and input and output filter quantity. However, there is another distinguishing factor: with these parameters being equal, the depthwise convolutions demonstrate their higher efficacy concerning the standard convolutions. This is visible by comparing the time’s inference of layer 0 (standard) with layer 1 (depthwise) or layer 5 (depthwise) with layer 6 (standard) and 9 (depthwise) and 10 (standard). These same layers couples represent the principal dimensionality redundancies of the best trade-off MobileNet structure. In this way, the executed pruning steps not only have focused on the critical time inference net bottlenecks but even on the layer with feature maps dimensionality already present in the architecture. However, the same approach has not been implemented for layer 2, where no redundancy was highlighted. The last remaining peak was represented by layer 16: to reduce successfully the pooling impact over time, the strides of layer 15 have been increased from (1 × 1) to (2 × 2).

**Figure 2:**
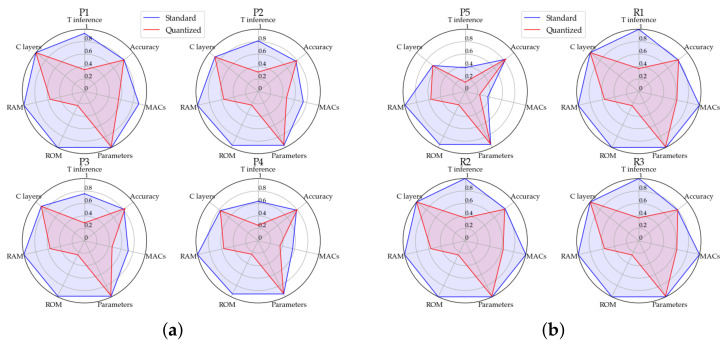
Optimization tests over best trade-off. P(i) means Pruning test while R(i) means Regularization tests, both executed on Keras (on blue) and quantized TensorFlow lite (on red) versions. (**a**) P1–P4 tests. (**b**) P5, R1–R3 tests.

Figure 2 helps to understand how each optimization technique improves several study parameters. Except for accuracies, that have values already ∈[0,1], each of these is normalized respect to all the 16 tests executed (8 of Keras and 8 of tflite) plus the best trade-off, namely starting point. The first key to the reading of this Figure 2 regards the pruning tests. The first five blue charts highlight remarkable progressive savings in terms of time inference and MAC operations, which reach 61.9% and 62.6%, respectively in P5, with a decrease of 6 C layers on the structure. Memory remains almost invariants to pruning applications, as well as accuracies evaluated on a random portion of 500 elements go from 0.832 of the starting point to 0.842 of P5, highlighting performance improvement in parallel to that computational. The three R1–R3 regularization tests have been carried out by adding definite values of L2 regularization following some state of art experiments and reporting only the interesting results among all the executions. In the study case, the weights regularization has been applied on dense layer 17, while the activations regularization on layer 15. The performances of these three tests are 0.822, 0.816, and 0.796, with respect to the 0.800 start accuracy on all the 6000 test set images. None of the computational parameters has been worsened by this technique, as can be seen on last three blue charts of Figure 2.

A further reading key of Figure 2 is represented by the “standard” and “quantized” charts. The gain on parameters, such as MACs, RAM, and ROM memories, and time inference, for both pruning and regularization techniques, is visible. Table 4 quantifies each variation in terms of means and standard deviations, normalized to the parameter’s maximum values over the whole of the represented tests. Remarkable mean decreases of 73.3% and 43.2% are highlighted by ROM and RAM values, respectively, that establish very low variances over the tests. MAC operations meanly enhance by 29.3%, but most of all 8-bit quantization reduces tests time inference by 50.9±13.4%, by meanly losing only the 0.002% in accuracy terms, becoming a key technique for hard real-time application. The last expected data are the invariance of C layers to quantization.

### 5.3. Explanation

**Figure 3:**
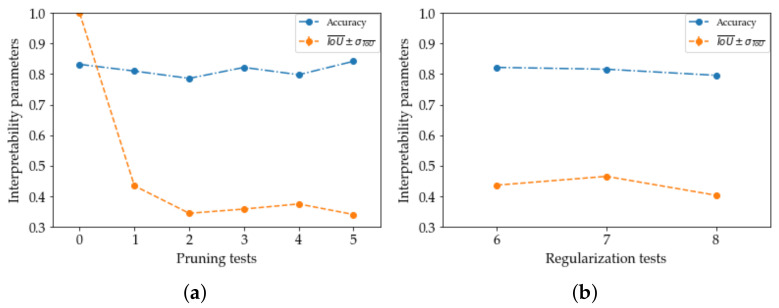
Interpretability results from over-optimization tests: accuracy and intersection over union trends. (**a**) P1–P5 tests. (**b**) R1–R3 tests.

Figure 3a,b show accuracies and intersections over unions for each test, for pruning and regularization, respectively. These graphs indicate two events: firstly, the trends of accuracy and IoU are largely similar to each other. Exceptions are steps P3 to P5, where trends are opposites. On all tests small variations are outlined, being able to observe substantial stability of both performance and interpretability factors. Table 5 reports these aspects for each execution. The low variances of these parameters between the eight tests mean not only having a certain safety margin in correct classification, but even LIME criteria remain the same for most of the test set images explainers. Finally, another aspect is represented by the low average value of IoU over the eight tests, equal to 0.394. Investigating a typical human detection problem through a classification task may involve not so high IoU values: LIME superpixels, differently by detection boxes, cannot isolate people one by one but act, including the densest areas of the image, without geometrical restrictive rules. This implies that the size that the superpixels union area of the two classifiers can reach over is large. Consequently, there are low values of IoU.

## 6. Conclusions

From an industrial point of view, the possibility to obtain immediately all the computational information regarding a Neural Network is the key to cutting down the time of production, without the necessity to deploy a Neural Network to low-level language manually. As a consequence of this, there is more margin to test a major number of algorithms.

Even for the optimization step, especially for pruning, the greatest advantage is represented by the possibility to analyze at layer by layer the time inference for each Neural Network, establishing a unique method to understand which layers to cut. Moreover, the same type of analysis for the other parameters suggests different procedures yet not developed, as the performance maintenance aiming at MACs or memories minimization: might be very useful in all those cases where more NNs must run simultaneously on the same board.

The optimization techniques conduct completely different impacts, especially pruning and quantization. The former greater earnings are represented by MACs and time inference, helping to reach real-time system production, but not necessarily decisively improving memories. On the contrary, quantization to 8-bit primarily helps to reduce Flash, RAM, and time inference, allowing not only real-time execution but even freeing space for running other Neural Networks. The improvement of MACs, in this case, is secondary but still consistent.

About the third step of the method, normally LIME allows only analyses on a single image whereas it has a limited explanation power on the whole dataset analyzed. Starting from this issue, the explanation step developed is one of the first methodologies able to guarantee global information by the whole dataset about image classification criterion expanding the single image interpretability concept.

Starting from deepening the method and offering a basic example of crowd counting, several tasks wherein AI and embedded systems meet themself could be deepened in the future with the method shown: detection of abnormal events [10], such as people aggressions, thefts, and objects loss; characterization of human gait [11]; congestion analysis; people identification; gender classification; pedestrian detection in urban scenes; and fall detection for the elderly, as an increasingly important field given the rise in life expectancy and the aging of populations.

In conclusion, the proposed method allows us to experiment with optimization techniques in a very easy way and to port the best Neural Network solution among the whole tests without writing any type of C code. The universality of what is presented admits to extending the same procedure for whatever computer vision task, microcontroller and also microprocessor board, and Deep Neural Network choice without any dramatic changes in the guideline, thus valid for most of the NNs implementations on edge devices.

## Figures and Tables

**Table 1:** Complete MobileNet architecture for binary classification. Dw Conv and FC indicate depthwise convolutions and fully connected, α the width multiplier, and ρ the resolution multiplier.

	Type/Stride	Filter Shape	Input Size
	Conv/s2	3 × 3 × 3 × 32·α	224·ρ × 224·ρ × 3
	Dw Conv/s1	3 × 3 × 32·α	112·ρ × 112·ρ × 32·α
	Conv/s1	1 × 1 × 32·α × 64·α	112·ρ × 112·ρ × 32·α
	Dw Conv/s2	3 × 3 × 64·α	112·ρ × 112·ρ × 64·α
	Conv/s1	1 × 1 × 64·α × 128·α	56·ρ × 56·ρ × 64·α
	Dw Conv/s1	3 × 3 × 64·α	56·ρ × 56·ρ × 128·α
	Conv/s1	1 × 1 × 128·α × 128·α	56·ρ × 56·ρ × 128·α
	Dw Conv/s2	3 × 3 × 128·α	56·ρ × 56·ρ × 128·α
	Conv/s1	1 × 1 × 128·α × 256·α	28·ρ × 28·ρ × 128·α
	Dw Conv/s1	3 × 3 × 256·α	28·ρ × 28·ρ × 256·α
	Conv/s1	1 × 1 × 256·α × 256·α	28·ρ × 28·ρ × 256·α
	Dw Conv/s2	3 × 3 × 256·α	28·ρ × 28·ρ × 256·α
	Conv/s1	1 × 1 × 256·α × 256·α	28·ρ × 28·ρ × 256·α
5×	Dw Conv/s1	3 × 3 × 512·α	14·ρ × 14·ρ × 512·α
	Conv/s1	1 × 1 × 512·α × 512·α	14·ρ × 14·ρ × 512·α
	Dw Conv/s2	3 × 3 × 512·α	14·ρ × 14·ρ × 512·α
	Conv/s1	1 × 1 × 512·α × 1024·α	7·ρ × 7·ρ × 1024·α
	Dw Conv/s2	3 × 3 × 1024·α	7·ρ × 7·ρ × 512·α
	Conv/s1	1 × 1 × 1024·α × 1024·α	7·ρ × 7·ρ × 1024·α
	Avg Pool/s1	7·ρ × 7·ρ	7·ρ × 7·ρ × 1024·α
	FC/s1	1024·α × 2	1024·α × 2
	Softmax	Classifier	1 × 1 × 2

**Table 2:** Computational and performance aspects of the tested RGB MobileNets.

Neural Networks	Parameters	ROM [kB]	RAM [kB] = IN + NET + OUT	MACs	Acc [ADIM]
Deep 128 α50	819,618	3.13 MB	733.45 = 196.81 + 536.83 + 8B	49,486,206	0.880
Shallow 128 α50	475,811	1.82 MB	733.45 = 196.81 + 536.83 + 8B	27,611,533	0.774
Deep 160 α25	213,586	823.63	724.62 = 307.20 + 417.41 + 8B	21,456,078	0.822
Deep 128 α25	213,586	834.32	465.03 = 196.61 + 268.41 + 8B	13,733,070	0.812
Shallow 160 α25	123,346	481.82	724.62 = 307.20 + 417.41 + 8B	12,558,798	0.841
Shallow 128 α25	123,346	476.13	465.03 = 196.61 + 268.41 + 8B	8,038,350	0.800

**Table 3:** Best trade-off method application over the two possible RGB choices.

Neural Networks	Acc [ADIM]	$\bar{T_{\inf}}\;\pm \;\sigma_{\;\bar{T_{\inf}}}$ [ms]	$\bar{\beta }\;\pm \;\sigma_{\;\bar{\beta }}$ [ADIM]
Deep 128 α25	0.812	430.911±1.468	32.823±2.398
Shallow 128 α25	0.800	285.873±0.728	30.610±2.328

**Table 4:** Relative gain for each investigation parameter due to quantization effect in terms of mean and standard dev. over the whole optimization tests.

Investigation Parameters	$\bar{\%}\pm \sigma_{\;\bar{\%}}$
Time inference	0.509±0.134
Accuracy	−0.002±0.001
MACs	0.293±0.082
Number of Pars	0.011±0.001
ROM	0.733±0.017
RAM	0.432±0.003

**Table 5:** Explainability trends: accuracy and IoU (in terms of mean and standard error) on each pruning and regularization test on 500 images test set.

Test	Accuracy	$\bar{\mathrm{IoU}}\pm \sigma_{\;\bar{\mathrm{IoU}}}$
P1	0.810	0.434±0.009
P2	0.786	0.344±0.009
P3	0.822	0.357±0.009
P4	0.798	0.374±0.001
P5	0.842	0.340±0.009
R1	0.822	0.436±0.010
R2	0.816	0.466±0.009
R3	0.796	0.403±0.009

## Data Availability

The FDST (Fudan-ShanghaiTech) dataset used is downloadable from https://paperswithcode.com/dataset/fdst.
